# OncoUroMiR: Circulating miRNAs for Detection and Discrimination of the Main Urological Cancers Using a ddPCR-Based Approach

**DOI:** 10.3390/ijms241813890

**Published:** 2023-09-09

**Authors:** José Pedro Sequeira, Daniela Barros-Silva, Patrícia Ferreira-Torre, Sofia Salta, Isaac Braga, João Carvalho, Rui Freitas, Rui Henrique, Carmen Jerónimo

**Affiliations:** 1 Cancer Biology & Epigenetics Group, Research Center of IPO Porto (CI-IPOP)/RISE@CI-IPOP (Health Research Network), Portuguese Oncology Institute of Porto (IPO Porto)/Porto Comprehensive Cancer Center Raquel Seruca (Porto.CCC), R. Dr. António Bernardino de Almeida, 4200-072 Porto, Portugal; jose.leite.sequeira@ipoporto.min-saude.pt (J.P.S.); daniela.barros.silva94@gmail.com (D.B.-S.); patricia.torre@ipoporto.min-saude.pt (P.F.-T.); sofia.salta@ipoporto.min-saude.pt (S.S.); isaac.braga@ipoporto.min-saude.pt (I.B.); joao.m.carvalho@ipoporto.min-saude.pt (J.C.); antoniofreitas@ipoporto.min-saude.pt (R.F.); henrique@ipoporto.min-saude.pt (R.H.); 2 Doctoral Programme in Biomedical Sciences, ICBAS-School of Medicine & Biomedical Sciences, University of Porto, Rua Jorge Viterbo Ferreira 228, 4050-513 Porto, Portugal; 3 Doctoral Programme in Molecular Pathology and Genetics, ICBAS-School of Medicine & Biomedical Sciences, University of Porto, Rua Jorge Viterbo Ferreira 228, 4050-513 Porto, Portugal; 4 Department of Urology & Urology Clinics, Portuguese Oncology Institute of Porto (IPOP), R. Dr. António Bernardino de Almeida, 4200-072 Porto, Portugal; 5 Doctoral Programme in Medical Sciences, ICBAS-School of Medicine & Biomedical Sciences, University of Porto, Rua Jorge Viterbo Ferreira 228, 4050-513 Porto, Portugal; 6 Department of Pathology, Portuguese Oncology Institute of Porto (IPO Porto)/Porto Comprehensive Cancer Center Raquel Seruca (Porto.CCC), R. Dr. António Bernardino de Almeida, 4200-072 Porto, Portugal; 7 Department of Pathology and Molecular Immunology, ICBAS–School of Medicine and Biomedical Sciences, University of Porto, Rua Jorge Viterbo Ferreira 228, 4050-513 Porto, Portugal

**Keywords:** ddPCR, biomarkers, circulating miRNAs, prostate cancer, renal cancer, bladder cancer, detection

## Abstract

The three most common genitourinary malignancies (prostate/kidney/bladder cancers) constitute a substantial proportion of all cancer cases, mainly in the elderly population. Early detection is key to maximizing the patients’ survival, but the lack of highly accurate biomarkers that might be used through non-/minimally invasive methods has impaired progress in this domain. Herein, we sought to develop a minimally invasive test to detect and discriminate among those urological cancers based on miRNAs assessment through ddPCR. Plasma samples from 268 patients with renal cell (RCC; *n* = 119), bladder (BlCa; *n* = 73), and prostate (PCa; *n* = 76) carcinomas (UroCancer group), and 74 healthy donors were selected. Hsa-miR-126-3p, hsa-miR-141-3p, hsa-miR-153-5p, hsa-miR-155-5p, hsa-miR-182-5p, hsa-miR-205-5p, and hsa-miR-375-3p levels were assessed. UroCancer cases displayed significantly different circulating hsa-miR-182-5p/hsa-miR-375-3p levels compared to healthy donors. Importantly, the hsa-miR-155-5p/hsa-miR-375-3p panel detected RCC with a high specificity (80.54%) and accuracy (66.04%). Furthermore, the hsa-miR-126-3p/hsa-miR-375-3p panel identified BlCa with a 94.87% specificity and 76.45% NPV whereas higher hsa-miR-126-3p levels were found in PCa patients. We concluded that plasma-derived miRNAs can identify and discriminate among the main genitourinary cancers, with high analytical performance. Although validation in a larger cohort is mandatory, these findings demonstrate that circulating miRNA assessment by ddPCR might provide a new approach for early detection and risk stratification of the most common urological cancers.

## 1. Introduction

Urological cancers, including those of the prostate (PCa), kidney, and bladder (BlCa), are a major cause of cancer-related morbidity and mortality [1,2]. Whereas PCa is the most frequent cancer among men, BlCa is the second-most frequent malignancy of the urinary tract, and renal cell carcinoma (RCC), although less incident, is the most lethal [1,3]. Altogether, they are the second most incident and third deadliest group of neoplasms, worldwide [1,4,5]. Their rising incidence, as well as the economic burden associated with diagnosis, treatment, and follow-up procedures, constitute a major challenge and concern in healthcare [6,7,8].

Urological cancers are mostly clinically silent at early stages, when curative therapy is more likely to be successful [9]. At present, however, only a handful of “screening” tests are available, and none address kidney cancer [10]. PCa is the only one with a widely used screening test based on serum prostate specific antigen (PSA) levels. However, using the most common cutoff value, serum PSA sensitivity for PCa detection does not exceed 21% [11,12]. For BlCa, urine cytology is often used to check for the presence of cancer cells, but has a significant false-negative rate, especially for low-grade carcinoma (10–50% accuracy) [13]. Considering the complexity of this scenario, new biomarkers tools are warranted for sensitive and accurate early detection, saving patient lives, and ensuring high quality of life.

Body fluids (e.g., blood and urine) are receiving special attention as a source of biomarkers in oncology, and circulating miRNAs are promising biomarkers in this context [14,15,16,17,18]. These small RNAs, with ~20 nucleotides, are involved in many vital functions, like cell differentiation/growth, and in tumorigenesis [16]. Indeed, dysregulated miRNA levels have been implicated in cancer development, including urological cancers [19,20,21,22,23]. Recent data indicate that microRNAs (miRNAs) might be invaluable biomarkers for detection, prognostication, and follow-up of urological cancers [18,24,25,26]. Moreover, the quality and quantity of miRNAs in circulation are essential for cancer cell communication and may be easily detected using conventional molecular biology techniques [27]. Therefore, considerable effort has been made to identify suitable cell-free miRNAs as potential non-invasive biomarkers in several malignancies [15]. As demonstrated in previous publications, the five circulating miRNAs analyzed in this study (hsa-miR-126-3p, hsa-miR-141-3p, hsa-miR-155-5p, hsa-miR-182-5p, and hsa-miR-375-3p) have been implicated in cancer. Specifically, an oncomiR role has been assigned to hsa-miR-155-5p and hsa-miR-182-5p, both evaluated in plasma, in RCC and PCa, respectively [25,28]. Moreover, plasma-circulating hsa-miR-126-3p and serum-circulating hsa-miR-141-3p enabled the identification of the most common RCC subtype, clear cell renal cell carcinoma [25,29]. Furthermore, plasma-circulating hsa-miR-375-3p was not only able to identify PCa metastasis but was also, together with plasma-circulating hsa-miR-141-3p, a predictor of progression in metastatic castration-resistant PCa [28,30].

The priority now is to design a methodological pipeline enabling miRNAs detection and quantification for optimal biomarker performance. However, low plasma sample input and the definition of the best normalizer constitutes barriers to progress in this area [25,26,31,32]. Quantitative real-time PCR (qRT-PCR), a routinely available technique, has been the most widely used method in published studies [15,16,17]. Nonetheless, inconsistent results are common owing mostly to the diversity of normalizers employed, impairing comparative studies and raising doubts about the most suitable normalizer to be used in circulating miRNAs assays [25]. Although miRNAs abundance in plasma is rather stable, sensitive and reproducible detection methods remain a critical issue that impairs their performance as biomarkers.

Notwithstanding the above-referenced limitations and challenges, circulating miRNAs have the potential to become effective urological cancer biomarkers for use in at-risk populations. Thus, the main objective of this study was to develop a test to accurately detect and discriminate among the three most common genitourinary cancers (PCa, BlCa, and RCC) based on a panel of plasma-circulating miRNAs, assessed through droplet digital PCR (ddPCR).

## 2. Results

### 2.1. Candidate miRNAs’ Selection

**Figure 1:**
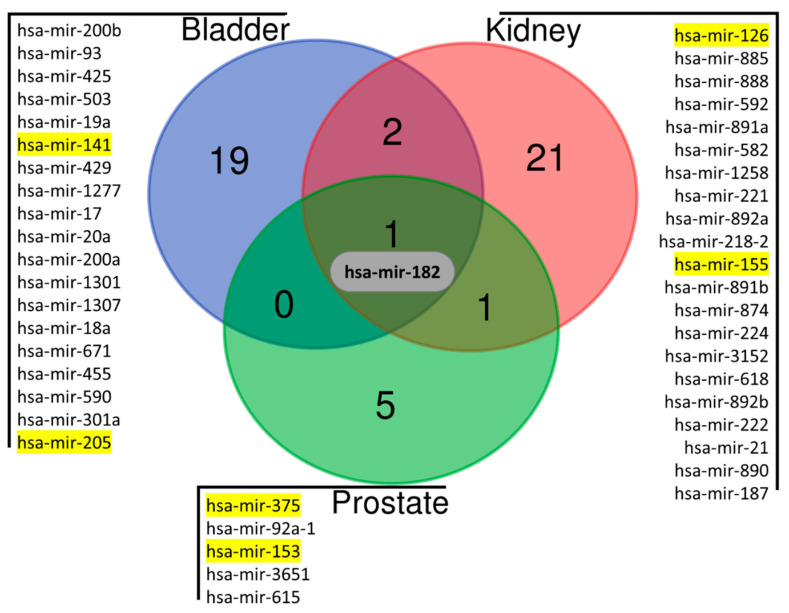
Venn diagram depicting miRNA candidates identified using OncoMiR Cancer Database (https://www.oncomir.umn.edu/omcd/ (accessed on 1 August 2022). Venn diagram was constructed using a web-based tool (https://bioinformatics.psb.ugent.be/webtools/Venn/ (accessed on 1 August 2022). The miRNAs candidates represented in yellow were selected for the discrimination of each urological cancer.

A panel of potentially useful and informative miRNAs was selected by systematic comparative analyses of urological cancer tissues’ miRNA levels accessed from a freely available web-based repository (https://www.oncomir.umn.edu/omcd/, accessed on 1 August 2022) and restricting the analysis to ratios of the highest levels/lowest levels that were higher than 5 (tumor tissue vs. normal tissue) and higher than 2 (tumor tissue vs. all tumor tissues—urological cancers, and breast/colorectal/lung cancers), with a *p*-value threshold of 0.001. In total, significantly high levels of 49 miRNAs were found in urological cancers, based on RNA sequencing (RNA-Seq) data extracted from those datasets. Out of the 49 candidate miRNAs with significantly higher levels, miR-182-5p was, in fact, simultaneously upregulated in the three most prevalent urological cancers: prostate, bladder, and kidney cancers (Figure 1). Besides urological cancer identification, we also looked for specific candidate miRNAs that would enable discrimination among those three cancers. Remarkably, 21 miRNAs were found to be specifically upregulated in kidney cancer compared to prostate and bladder cancers. Furthermore, 19 and 5 miRNAs were found to be specifically upregulated in bladder and prostate cancers, respectively.

Considering these results and following a detailed literature review, a panel of seven candidate miRNAs (hsa-miR-126-3p, hsa-miR-141-3p, hsa-miR-153-5p, hsa-miR-155-5p, hsa-miR-182-5p, hsa-miR-205-5p, and hsa-miR-375-3p) was selected for further testing using plasma samples of patients harboring a urological cancer (Figure S1). Specifically, one miRNA (hsa-miR-182-5p) that displayed high levels in the three tumor types, and two miRNAs for each type of tumor were selected. For BlCa, the top three miRNAs, hsa-miR-141-3p, hsa-miR-205-5p, and hsa-miR-93 (with the greatest differences in the TCGA data), were selected, but hsa-miR-93 was not further analyzed due to the lack of information in the literature. The same approach was used for PCa, with two miRNAs (hsa-miR-375-3p and hsa-miR-153-5p) selected for subsequent analysis, while hsa-miR-92a-1 was dropped. Finally, for RCC detection, three miRNAs that showed significant results in the in silico analysis (hsa-miR-126-3p, hsa-miR-155-5p, and hsa-miR-21-5p) were selected. However, previous studies demonstrated that hsa-miR-21-5p levels were highly biased by platelets in which this miRNA is present at high levels [25,33,34]. Moreover, the levels of the significantly different miRNAs (hsa-miR-126-3p, hsa-miR-155-5p, hsa-miR-183-5p, and hsa-miR-375-3p) were evaluated in the three most common non-urological cancers (lung, colorectal, and breast cancers) through in silico analysis (restricting the analysis to ratios of altered levels higher than 5 and a *p*-value threshold of 0.001) (Figure S2). Hsa-miR-375-3p showed significantly higher levels in invasive breast carcinoma, and lower levels in colorectal cancer. Moreover, hsa-miR-182-5p presented higher levels in lung, colon, and breast cancer tissues than in the respective normal tissues. The other selected miRNAs did not have altered levels in these non-urological cancers compared to normal tissues. These results are in line with the reported miRNAs variability in different tumors [25,35]. Nevertheless, this does not hamper the results of our study since our test is intended for patients with urological symptoms.

### 2.2. Patients’ Characteristics

The relevant clinical and pathological parameters of the patients’ cohort are depicted in Table 1. Potential confounder variables were taken into consideration during the study set up. For instance, approximately 60% of all cases included were at an early stage/grade since our purpose was to set up a screening test for this condition. Except for PCa, the proportion of male subjects in the study was 76% (147 male/192 RCC and BlCa cases), as RCC and BlCa are about 3 times more prevalent in men than in women, which closely fits with our cohort. The men/women ratios were kept in mind for healthy blood donors (HD) group selection as well. Regarding age parameters, urological cancer is most common in elderly people, and the median age of the patients was 66 years old (with a 75th percentile of 73 years old).

### 2.3. MiRNAs Levels in Plasma of Tumors and Healthy Donors

Tissues and plasma samples often exhibit different miRNAs levels, suggesting that results from studies with different starting materials may not be comparable. Thus, the candidate tissue miRNAs were tested by ddPCR using plasma samples. All the selected miRNAs were detected in circulation except hsa-miR-153-5p and hsa-miR-205-5p (Figure S3). Moreover, to ensure that the miRNAs identified were representative of those found in a broad set of plasma samples, we discarded miRNAs with a low abundance. For instance, miR-141-3p levels were very low in plasma compared to normal samples and no statistically significant differences were found between the tested groups for this miRNA.

**Figure 2:**
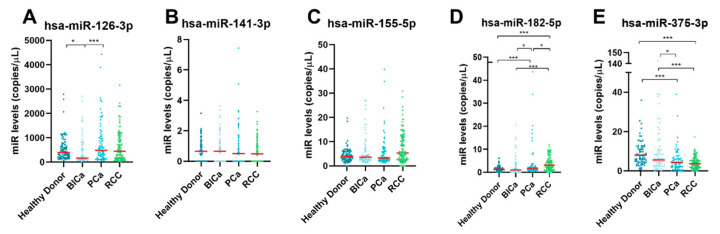
Dot-plots of hsa-miR-126-3p (**A**), hsa-miR-141-3p (**B**), hsa-miR-155-5p (**C**), hsa-miR-182-5p (**D**), and hsa-miR-375-3p (**E**) levels in PCa patients, BlCa patients, RCC patients, and healthy donors. The horizontal red lines represent miRNAs’ median levels. Abbreviations: BlCa—bladder cancer, PCa—prostate cancer, RCC—renal cell carcinoma; *—*p*-value ≤ 0.05, ***—*p*-value ≤ 0.001.

The initial comparisons of the plasma-circulating miRNAs levels among all the groups are illustrated in Figure 2. Circulating miR-126-3p levels were lower in BlCa than in HD samples and PCa patients, whereas circulating miR-182-5p levels were exceptionally higher in RCC patients compared with all the other groups. In contrast, circulating miR-375-3p was lower in all cancer samples compared to HD. Moreover, no significant differences were found in miRNA levels among advanced stages (Figure S4). Nonetheless, circulating miR-182-5p, miR-375-3p, and miR-126-3p levels were significantly different between early-stage RCC and early-stage BlCa, and between early-stage PCa and early-stage BlCa (Figure S5). Of note, early-stage RCC displayed higher miR-182-5p circulating levels than early-stage PCa.

**Figure 3:**
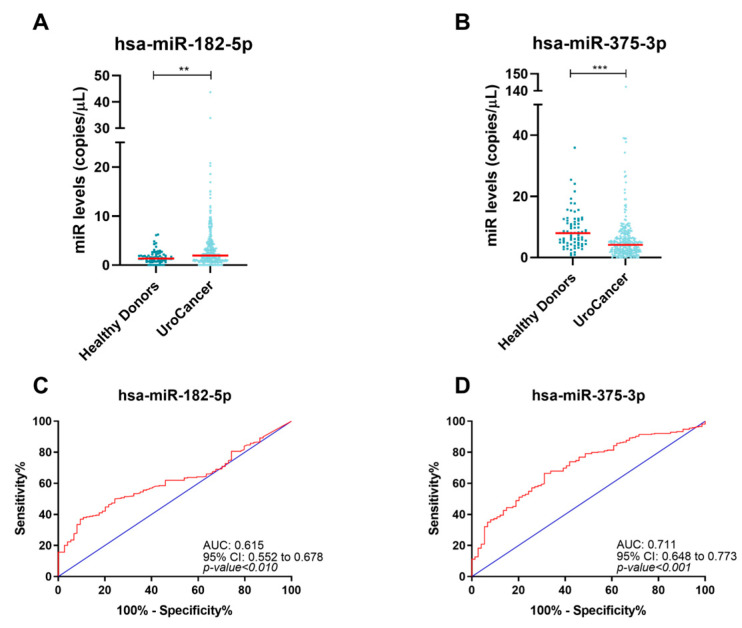
Dot-plots of hsa-miR-182-5p (**A**) and hsa-miR-375-3p (**B**) levels in urological cancers and healthy donors. The horizontal red lines represent miRNAs’ median levels. Receiver operating characteristic curves of hsa-miR-182-5p (**C**) and hsa-miR-375-3p (**D**) for detection of urological cancers. The blue and red lines indicate the reference line and the identity line for each miRNA, respectively. **—*p*-value ≤ 0.01, ***—*p*-value ≤ 0.001.

When PCa, BlCa, and RCC were grouped into a single urological cancer group, two out of the five tested circulating miRNAs—miR-182-5p and miR-375-3p—differed significantly between the cancer group and HD (Figure 3A,B and Figure S6). Meanwhile, miR-182-5p levels were higher in urological cancers (*p*-value = 0.002), and miR-375-3p levels were lower (*p*-value < 0.001) compared to HD.

Subsequently, the potential of these two miRNAs for detecting urological cancers was assessed by ROC analysis (Figure 3C,D and Table 2). The test was considered positive when miR-182-5p levels were higher or when miR-375-3p levels were lower than the cut-off determined by the ROC curve analysis. Although the individual sensitivity of each miRNA was quite modest, when combined in a panel (at least one positive), the test detected 65% of the cancers with an accuracy above 68%, resulting in an 81% specificity and an impressive 93% PPV.

Interestingly, the same miRNAs could discriminate early-stage (Figure S7) and advanced-stage tumors from healthy donors (Figure S8), emphasizing the potential of miR-182-5p and miR-375-3p for detecting the three most common urological cancers. The combination of both biomarkers was able to detect 78% of early-stage tumors and 89% of advanced-stage tumors, with an accuracy above 72% in both scenarios (Tables S1 and S2).

### 2.4. Circulating miRNAs Performance in Discriminating RCC Patients from Other Urological Cancer Patients

**Figure 4:**
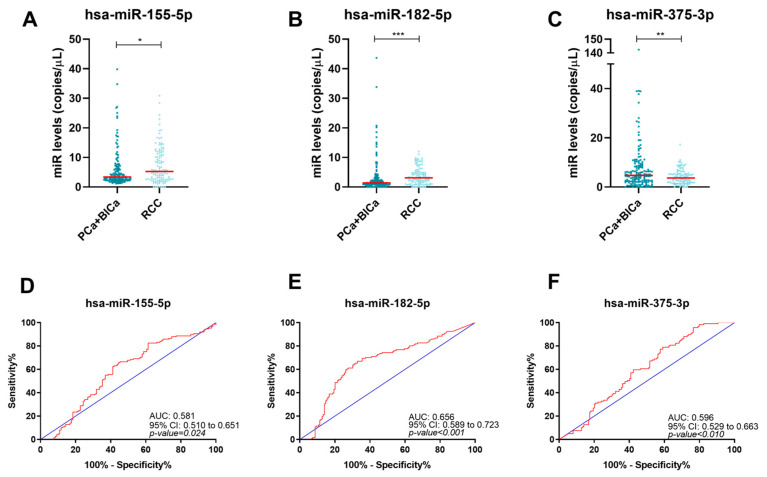
Dots-plots of hsa-miR-155-5p (**A**), hsa-miR-182-5p (**B**), and hsa-miR-375-3p (**C**) levels in RCC and BlCa/PCa group. The horizontal red lines represent miRNAs’ median levels. Receiver operating characteristic curves of hsa-miR-155-5p (**D**), hsa-miR-182-5p (**E**), and hsa-miR-375-3p (**F**) for RCC detection. The blue and red lines indicate the reference line and the identity line for each miRNA, respectively. Abbreviations: BlCa—bladder cancer, PCa—prostate cancer, RCC—renal cell carcinoma, *—*p*-value ≤ 0.05, **—*p*-value ≤ 0.01, ***—*p*-value ≤ 0.001.

Then, a comparison between RCC and a group comprising the other types of urological cancers (BlCa and PCa) was performed. The levels of three out of the tested miRNAs—hsa-miR-182-5p, hsa-miR-155-5p, and hsa-miR-375-3p—were significantly different in RCC (*p*-value < 0.001, *p*-value = 0.024 and *p*-value = 0.007, respectively) compared with BlCa and PCa (Figure 4 and Figure S9). Circulating miR-182-5p and miR-155-5p levels were higher, and miR-375-3p levels were lower in RCC. Although miR-182-5p, miR-155-5p, and miR-375-5p could discriminate RCC from the other urological malignancies with a good negative predictive value (NPV > 65% for both miRNAs) (Table 3), we looked for a multi-marker panel enabling urological cancer discrimination. The overall biomarker combination was only considered positive if all the included miRNAs were positive. The cutoffs used in this analysis were established according to the ROC curve. The best performing biomarker combination comprised miR-155-5p and miR-375-5p, thus excluding miR-182-5p. In fact, the two-biomarker panel depicted improved diagnostic performance, with an 80.5% specificity. Thus, combined analysis of circulating miR-155-5p and miR-375-3p was able to discriminate RCC patients from PCa and BlCa patients with a low false-positive rate.

In the same vein, the levels of four out of the tested miRNAs—hsa-miR-126-3p, hsa-miR-182-5p, hsa-miR-155-5p, and hsa-miR-375-3p—were significantly different in early-stage RCC (*p*-value = 0.034, *p*-value < 0.001, *p*-value = 0.016 and *p*-value = 0.048, respectively) compared with early-stage BlCa or PCa (Figure S10). Moreover, high hsa-miR-182-5p levels and low hsa-miR-141-3p levels were found in advanced-stage RCC (Figure S11). Likewise, the panel comprising miR-375-3p and miR-155-5p accurately detected early-stage RCC (70.12%) (Table S3), whereas miR-141-4p identified advanced-stage RCC with a 60.81% sensitivity and 60.42% specificity (Table S4).

### 2.5. Circulating miRNAs Performance in Discriminating BlCa Patients from Other Urological Cancer Patients

**Figure 5:**
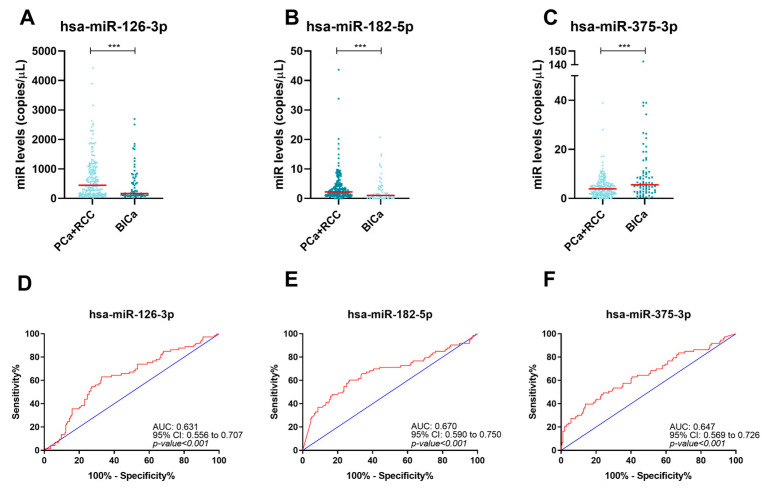
Dots-plots of hsa-miR-126-3p (**A**), hsa-miR-182-5p (**B**), and hsa-miR-375-3p (**C**) levels in BlCa and RCC/PCa group. The horizontal red lines represent miRNAs’ median levels. Receiver operating characteristic curves of hsa-miR-126-3p (**D**), hsa-miR-182-5p (**E**), and hsa-miR-375-3p (**F**) for BlCa detection. The blue and red lines indicate the reference line and the identity line for each miRNA, respectively. Abbreviations: RCC—renal cell carcinoma, BlCa—bladder cancer, PCa—prostate cancer; ***—*p*-value ≤ 0.001.

Despite circulating miR-375-3p levels being lower in cancer samples compared to HD, its levels were strikingly higher in BlCa compared to PCa and RCC (Figure 2E and Figure 5C). Inversely, circulating hsa-miR-126-3p (Figure 2A and Figure 5A) and hsa-miR-182-5p (Figure 2D and Figure 5B) levels were significantly lower in BlCa compared to PCa and RCC plasma samples. The best combination was achieved by combining hsa-miR-126-3p and hsa-miR-375-3p. Once again, a biomarker test was set up comprising both miRNAs and was considered positive when circulating miR-375-3p levels were higher and circulating miR-126-3p levels were lower than the cutoff established by the ROC curve. This panel discriminated BlCa from PCa and RCC with a high specificity (94.87%) and accuracy (75%) (Table 4). The levels of the other tested miRNAs did not show statistically significant differences between the groups (Figure S12).

Although no significant differences were found among advanced-stage disease across the three cancer models, the same three miRNAs (hsa-miR-126-3p, hsa-miR-182-5p, and hsa-miR-375-3p) were able to discriminate early-stage BlCa from early-stage RCC/PCa (*p*-value < 0.001) (Figure S13). Indeed, the combination of hsa-miR-126-3p and hsa-miR-375-3p identified this set of tumors with a 95.58% specificity and 75.61% accuracy (Table S5).

### 2.6. Circulating miRNAs Performance in Discriminating PCa Patients from Other Urological Cancer Patients

**Figure 6:**
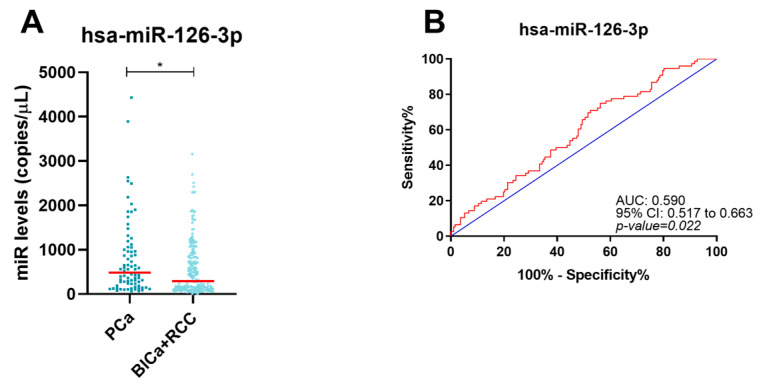
Dot-plot of hsa-miR-126-3p (**A**) in PCa and RCC/BlCa group. The horizontal red lines represent miRNAs’ median levels. Receiver operating characteristic curve of hsa-miR-126-3p (**B**) for PCa detection. The blue and red lines indicate the reference line and the identity line for each miRNA, respectively. Abbreviations: BlCa—bladder cancer, PCa—prostate cancer, RCC—renal cell carcinoma, *—*p*-value ≤ 0.05.

Finally, from the tested circulating miRNAs (Figure S14), circulating miR-126-3p was the only one that segregated PCa patients from the other urological cancer patients (Figure 6; *p*-value = 0.021) with a 81.5% NPV and 52.6% accuracy (Table 5). The same miRNA discriminated advanced-stage PCa from advanced-stage BlCa and RCC (*p*-value = 0.033) (Figure S15) with an 83.4% specificity and 71.57% accuracy (Table S6). Furthermore, early-stage PCa had lower hsa-miR-375-5p levels (*p*-value = 0.041) (Figure S16), identifying this set of tumors with a 60.4% sensitivity and 78.16% NPV (Table S7).

## 3. Discussion

Notwithstanding their biological and clinical diversity, PCa, BlCa, and RCC constitute a significant clinical challenge [7], and owing to their clinically silent development at early stages, only the use of molecular biomarkers might enable early cancer detection, reducing mortality and increasing quality of life for these patients. At-risk populations, such as elderly people, smokers, workers exposed to occupational risk factors, and people with a family history of cancer or inherited conditions, would greatly benefit from effective screening tools [4]. Nonetheless, few screening tests are available in clinical practice to uncover urological tumors at early stages, and their performance is unsatisfactory [10,11,12,36]. In the era of Precision Oncology, liquid biopsy techniques, especially the analysis of plasma circulating nucleic acids, represent a paradigm shift in the biomarkers arena, with considerable implications for clinical practice [16,37]. Indeed, DNA-based detection of malignancies from plasma and/or urine has been previously reported for kidney, bladder, and prostate cancer [38]. Circulating cell-free RNA may provide important information on the expression of various target genes non-invasively without the need for tissue biopsies [39]. Indeed, the abundance of cell-free miRNA has been measured in plasma and proposed as a source of novel, minimally invasive biomarkers for several diseases [40]. As reported by Syeda and colleagues, a wide variety of studies reported that aberrant miRNAs’ levels may be found during cancer development, reflecting oncogenic or tumor-suppressor behavior [41]. Moreover, miRNAs’ levels have been suggested as potential biomarkers for cancer diagnosis, prognostication, and prediction of response to therapy [16,41]. In this context, the development of a circulating biomarker panel that can be used in populations at risk of developing urological cancers could overcome the current limitations.

In this study, we used a ddPCR assay, which is a robust, precise, and sensitive technology that, contrarily to qRT-PCR, the gold standard for miRNAs quantification, obviates the use of normalization methods [26,42,43]. Recent data demonstrated that ddPCR possesses a good recovery of spike-in and, notably, sample detection with tumor burden representation even without a preamplification step. Moreover, ddPCR has lower intra- and inter-operator variability, which further supports its potential for biomarker investigation [26]. Moreover, Taylor et al. reported that in samples containing a low nucleic acid input (Cq > 29), ddPCR is highly recommended as it yields more accurate and reproducible results [43].

Overall, we found that patients from the UroCancer group displayed significantly different circulating levels of hsa-miR-182-5p/hsa-miR-375-3p compared to healthy donors, even when the analyses were restricted to early- or advanced-stage disease. Notably, the hsa-miR-155-5p/hsa-miR-375-3p panel detected RCC with a high specificity (80.54%) and reasonable accuracy (66.04%). Furthermore, the hsa-miR-126-3p/hsa-miR-375-3p panel identified BlCa patients with a 94.87% specificity and 76.45% NPV whereas hsa-miR-126-3p levels were higher in the PCa group. The good performance of the hsa-miR-155-5p/hsa-miR-375-3p panel for RCC identification underlines the important role of miRNAs as circulating biomarkers for RCC detection, as we and others have previously advocated [16,25]. Moreover, the BlCa results also show a high potential due to the similarity of the performance parameters compared with cytology, and with the advantage of being less operator-dependent [44,45,46,47,48]. Although the biomarker performance of miR-126-3p for PCa patient identification against other urological tumors was rather limited, the lower cancer specificity of the serum PSA test must be considered as well. Indeed, recent data from an analysis of 14,489 patients estimated a 93% sensitivity for serum PSA ≥ 4 ng/mL (95% CI 0.88, 0.96) but only 20% (95% CI 0.12, 0.33) specificity [49]. It should be acknowledged that some of the tested panels had low sensitivity (e.g., detection of BlCa vs. PCa + RCC: 21.92%), which constitutes a major limitation to its use in a routine clinical setting. Nonetheless, considering the suboptimal performance or unavailability of clinically validated early detection tools for PCa, BlCa, and RCC, we believe that the high specificity of the biomarkers (e.g., BlCa vs. PCa + RCC: higher than 94%) constitutes an encouraging result. Furthermore, it should be emphasized that in most of the biomarker panels analyzed, the accuracy exceeded 70%. Because the low sensitivity might be improved through repeated testing (i.e., increasing the frequency of testing time points), these biomarker panels may well surpass existing early detection tools and provide novel opportunities for integrated urological cancer screening strategies.

The assessment of miRNAs levels in plasma samples is challenging, with several conditions having the possibility of introducing bias. For instance, hemolysis alters plasma miRNA content and may confound biomarker discovery and assessment [50,51,52]. Thus, we established a verification step for hemolysis and excluded all cases in which it was apparent, since miRNA traces expressed in red blood cells are released by hemolysis. Moreover, circulating miRNA levels may be impacted by age and gender [53,54]. Differences in blood cell parameters, usually higher in men, might be explained by gender-related differences in miRNAs levels [55]. Nevertheless, since hemolyzed plasma samples were excluded from our analysis, only a minor effect should be expected. Yet, miR-155-5p, miR-126-3p, and miR-182-5p demonstrated an association with gender, with higher levels in females compared to males. This may be related to steroid sex hormones, such as estradiol and progesterone, which seem to directly regulate miRNAs levels and/or regulate the major enzymes involved in miRNA biogenesis [56]. Nevertheless, according to the literature, aging seems to be more strongly associated with circulating miRNAs levels than gender, since miRNAs’ upregulation mostly associates with increased age [55,57]. Concordantly, in our study, significant correlations were found between levels of some miRNAs and age, but, interestingly, the opposite trend was found for miR-126-3p and miR-375-3p.

One of the major limitations of the study was that candidate miRNAs’ selection was based on tissue samples profiles, which may not specifically mimic miRNAs levels in circulation, but the current lack of databases with miRNAs levels assessed from liquid biopsies prevented a more appropriate analysis. Furthermore, the use of plasma RNAs as biomarkers is often seen with some skepticism by experts in the field due to the short half-life in circulation [58]. However, a vast number of studies already demonstrated that miRNAs are remarkably resistant to RNase degradation and quite stable either because they are in protein-associated complexes or due to several RNA modifications [59,60]. The OncoUroMiR panel is intended for testing individuals with urological symptoms common to the three cancer types, including hematuria, dysuria, pelvic discomfort, and weak urine stream. Thus, in the future, larger prospective studies to validate these biomarker panels should be conducted to pave the way for the translation of these novel tools into clinical practice.

## 4. Materials and Methods

### 4.1. Plasma Samples Collection and Processing

Between 2015 and 2021, peripheral blood samples were collected into EDTA-containing tubes and processed within a 4 h timeframe. Plasma was separated by centrifugation at 2500× *g* for 30 min at 4 °C and subsequently stored at −80 °C in the institutional biobank until further usage. All plasma samples were inspected for hemolysis, as previously reported [25]. Of the 453 initially selected samples, 111 had an absorbance at 414 nm higher than 0.25 and were excluded. Thus, a set of plasma samples comprising 119 RCC, 73 BlCa, and 76 PCa cases, as well as 74 HD were further analyzed. Informed consent was obtained for all tested samples and the study received approval by the institutional Ethics Committee (Comissão de Ética para a Saúde—CES-IPOP 518/010, 216R/019, 107/020).

Relevant clinical and pathological data were retrieved from clinical charts and grouped in a pseudo-anonymized database constructed for the purposes of this study.

### 4.2. Bioinformatics Analysis for Selection of Candidate miRNAs

The in silico analysis was performed using data retrieved from the OncoMir Cancer Database [61], a repository that allows easy and systematic comparative analysis of miRNA sequencing data derived from cancer patients and organ-specific controls present in The Cancer Genome Atlas (TCGA) database. A selection of five urological cancer studies were included [bladder urothelial carcinoma (BLCA), kidney chromophobe (KICH), kidney renal clear cell carcinoma (KIRC), kidney renal papillary cell carcinoma (KIRP), and prostate adenocarcinoma (PRAD)]. Therefore, a total of 1765 cancer samples were used in the analysis: 28% from prostate, 23% from bladder, and 49% from kidney cancers.

The search for miRNA statistical results was accomplished based on specific defined thresholds. A *p*-value threshold of 0.001 was set, and a ratio for differential higher levels above 5 (tumor tissue vs. normal tissue) and higher than 2 (tumor tissue vs. all tumor tissues—urological cancers, and breast/colorectal/lung cancers) was considered.

### 4.3. RNA Isolation and Quantification of Circulating miRNAs’ Levels

Total RNA was isolated from plasma samples and reverse transcribed as previously described [25,26] using the Veriti™ thermocycler (Applied Biosystems™, Waltham, MA, USA) for spike-in (ath-miR-159a, ID 000338, mature miRNA sequence: UUUGGAUUGAAGGGAGCUCUA), and all miRNAs of interest [hsa-miR-126-3p (ID 002228, mature miRNA sequence: UCGUACCGUGAGUAAUAAUGCG), hsa-miR-141-3p (ID 000463, mature miRNA sequence: UAACACUGUCUGGUAAAGAUGG), hsa-miR-153-5p (ID 466540_mat, mature miRNA sequence: UCAUUUUUGUGAUGUUGCAGCU), hsa-miR-155-5p (ID 467534_mat, mature miRNA sequence: UUAAUGCUAAUCGUGAUAGGGGUU), hsa-miR-182-5p (ID 002334, mature miRNA sequence: UUUGGCAAUGGUAGAACUCACACU), hsa-miR-205-5p (ID 000509, mature miRNA sequence: UCCUUCAUUCCACCGGAGUCUG), and hsa-miR-375-3p (ID 000564, mature miRNA sequence: UUUGUUCGUUCGGCUCGCGUGA)].

A qualitative analysis of the spike-in ath-miR-159a was performed. Specifically, ath-miR-159a levels were determined by ddPCR. Indeed, when the artificial miRNA’s concentration was out of the range of the values obtained for all the other samples, RNA isolation was repeated of that given sample. For quantitative miRNA analysis, ddPCR analysis was performed for hsa-miR-126-3p, hsa-miR-141-3p, and hsa-miR-155-5p, as previously reported by our team [25]. Optimization for the other miRNAs was performed using the same ddPCR pipeline. Importantly, the cDNA volumes used included 2 µL (hsa-miR-205-5p), 4 µL (hsa-miR-182-5p), and 5 µL (hsa-miR-375-3p). Hsa-miR-153-5p and hsa-miR-205-5p were excluded from the analysis as no detectable levels were found in circulation (Figure S3). The ddPCR run with the following conditions: 95 °C 10 min, 50 cycles of 94 °C 30 s, and annealing temperature optimized using 1 min—ramp rate 2 °C/s—and 98 °C 10 min. The annealing temperature for the newly optimized miRNAs was 56 °C for hsa-miR-182-5p and hsa-miR-375-3p.

The limit of blank (LOB) and limit of detection (LOD) were calculated using 30 non-template controls according to Milbury et al. [62]. Positive control, no template control (NTC), and no enzyme control (NEC) were included in all cDNA synthesis and ddPCR steps. All samples were run in a single reaction for each target. Importantly, as we [16,25,26] and others [42,63] have previously reported, ddPCR is a technology based on Poisson distribution, and does not require a housekeeping/normalizer miRNA due to the absolute quantification capacity. This approach obviates the variability in miRNA/lncRNA/gene levels which are usually unstable in liquid biopsies, leading to biased results in qPCR. Currently, there is no consensus about which housekeeping should be used [16].

### 4.4. Statistical Analysis

The levels of each miRNA were compared between tumor types, and associations with clinicopathological features were also assessed. Tumors were categorized into early-stage vs. advanced-stage tumors according to the following criteria: early-stage RCC (Stage I + II) vs. advanced-stage RCC (Stage III + IV) [25]; early-stage PCa (ISUP 1 + 2) vs. advanced-stage PCa (ISUP 3 + 4 + 5) [64]; early-stage BlCa (Ta-T1; non-muscle-invase BlCa) vs. advanced-stage BlCa (T2-T4; muscle-invasive BlCa) [65]. The Kruskal–Wallis test was used for multiple comparisons, and pairwise comparisons were accomplished using the Mann–Whitney U test with Bonferroni’s correction.

Samples were categorized as positive or negative for each miRNA based on the cutoff values established using Youden’s J index [66,67], combining the highest sensitivity and specificity, through receiver operator characteristic (ROC) curve analysis. Validity estimates [sensitivity, specificity, positive predictive value (PPV), negative predictive value (NPV), and accuracy] were calculated to assess biomarker performance in detecting malignancy. True positive (TP) was defined as a cancer case which tested positive based on miRNA levels, whereas a true negative (TN) was defined as a control that tested negative based on miRNA levels. Moreover, a false negative (FN) was defined as a case that tested negative based on miRNA levels whereas a false positive (FP) was defined as a control that tested positive based on miRNA levels. Sensitivity, specificity, accuracy, positive predictive value (PPV), and negative predictive value (NPV) were calculated according to the formulas: sensitivity = TP/total number of cases (TP + FN); specificity = TN/total number of controls (TN + FP); accuracy = (TP + TN)/total number of samples (TP + TN + FP + FN); PPV = TP/total number of positive samples for each miRNA (TP + FP); NPV = TN/total number of negative samples for each miRNA (TN + FN).

To increase the detection performance, the panel comprising tumor vs. non-tumor sample comparisons was constructed considering a positive result when at least one target miRNA was considered positive in the individual analysis. Moreover, for tumor type discrimination, a result was considered positive when both miRNAs were positive in the individual analysis.

Two-tailed *p*-value calculations and receiver operating characteristic curve analysis was performed using SPSS 27.0 for Windows software (IBM-SPSS Inc., Chicago, IL, USA). All graphics were generated using GraphPad Prism 8.0 for Windows Software (GraphPad Software Inc., La Jolla, CA, USA). A *p*-value < 0.05 was considered statistically significant. A summary of the methods workflow is presented in Figure S17.

## 5. Conclusions

In brief, we demonstrated the feasibility of using a minimally invasive test based on a panel of circulating miRNAs, assessed by ddPCR, to detect and discriminate among the three most common urological cancers. A larger, multicentric study is required to further validate these findings. This strategy might contribute to establishing early detection programs for these urological malignancies, using minimally invasive sample collection and high-throughput assays, which are the key for their successful implementation in the clinic.

## Figures and Tables

**Table 1:** Clinicopathological data of the patient cohort and healthy donors used in this study.

**OncoUroMiR Cohort**	
Renal cell carcinoma (RCC, *n*)	119
Prostate carcinoma (PCa, *n*)	76
Bladder carcinoma (BlCa, *n*)	73
Healthy blood donors (HD, *n*)	74
**RCC (*n* = 119)**	
**Age [years (median, interquartile range)]**	64 (17)
**Gender (*n*)**	
Male	86/119 (72.3%)
Female	33/119 (27.7%)
**Stage (*n*)**	
I	57/119 (47.9%)
II	8/119 (6.7%)
III	42/119 (35.3%)
IV	12/119 (10.1%)
**ISUP Grade (*n*)**	
1	7/119 (5.9%)
2	44/119 (37.0%)
3	22/119 (18.5%)
4	10/119 (8.4%)
n.a.	36/119 (30.2%)
**PCa (*n* = 76)**	
**Age [years (median, interquartile range)]**	63 (8)
**ISUP Group Grade (*n*)**	
1	24/76 (31.6%)
2	24/76 (31.6%)
3	15/76 (19.7%)
4	6/76 (7.9%)
5	7/76 (9.2%)
**BlCa (*n* = 73)**	
**Age [years (median, interquartile range)]**	70 (15)
**Gender (*n*)**	
Male	61/73 (83.6%)
Female	12/73 (16.4%)
**T (*n*)**	
a	23/73 (31.5%)
1	28/73 (38.4%)
2	11/73 (15.1%)
3	4/73 (5.5%)
4	5/73 (6.8%)
x	2/73 (2.7%)
**N (*n*)**	
0	68/73 (93.2%)
1	2/73 (2.7%)
2	2/73 (2.7%)
3	1/73 (1.4%)
**M (*n*)**	
0	70/73 (95.9%)
1	3/73 (4.1%)
**Histological grade (*n*)**	
Low grade	26/73 (35.7%)
High grade	45/73 (61.6%)
Unknown	2/73 (2.7%)
**Carcinoma in situ (*n*)**	
No	59/73 (80.8%)
Yes	11/73 (15.1%)
Unknown	3/73 (4.1%)
**HD (*n* = 74)**	
**Age [years (median, interquartile range)]**	46 (9)
**Gender (*n*)**	
Male	47/74 (63.5%)
Female	27/74 (36.5%)

**Table 2:** Performance of hsa-miR-182-5p and hsa-miR-375-3p as biomarkers for urological cancer detection. Abbreviations: SE—sensitivity; SP—specificity; PPV—positive predictive value; NPV—negative predictive value.

miRNA	SE %	SP %	PPV %	NPV %	Accuracy %
**hsa-miR-182-5p**	36.94	90.54	93.40	28.39	48.54
**hsa-miR-375-3p**	36.94	90.54	93.40	28.39	48.54
**miRNA panel**	64.93	81.08	92.55	38.96	68.42

**Table 3:** Performance of hsa-miR-155-5p and hsa-miR-375-3p as biomarkers for RCC detection among urological tumors. Abbreviations: SE—sensitivity; SP—specificity; PPV—positive predictive value; NPV—negative predictive value.

miRNA	SE %	SP %	PPV %	NPV %	Accuracy %
**hsa-miR-155-5p**	58.82	61.74	55.12	65.25	60.45
**hsa-miR-375-3p**	78.99	40.94	51.65	70.93	57.84
**hsa-miR-182-5p**	67.23	67.11	71.94	71.94	67.16
**miRNA panel (hsa-miR-155-5p + hsa-miR-375-3p)**	47.90	80.54	66.28	65.93	66.04

**Table 4:** Performance of hsa-miR-126-3p, hsa-miR-182-5p, and hsa-miR-375-3p as biomarkers for BlCa detection. Abbreviations: SE—sensitivity; SP—specificity; PPV—positive predictive value; NPV—negative predictive value.

miRNA	SE %	SP %	PPV %	NPV %	Accuracy %
**hsa-miR-126-3p**	63.01	67.18	41.82	82.91	66.04
**hsa-miR-182-5p**	60.27	73.33	45.83	83.14	69.78
**hsa-miR-375-3p**	39.73	86.15	51.79	79.25	73.51
**miR Panel (hsa-miR-126-3p + hsa-miR-375-3p)**	21.92	94.87	61.54	76.45	75.00

**Table 5:** Performance of hsa-miR-126-3p as biomarker for PCa detection. Abbreviations: SE—sensitivity; SP—specificity; PPV—positive predictive value; NPV—negative predictive value.

miRNA	SE %	SP %	PPV %	NPV %	Accuracy %
**hsa-miR-126-3p**	75.00	43.75	34.55	81.55	52.61

## Data Availability

All data generated or analyzed during this study are included in this article.

## Supplementary Materials

The following supporting information can be downloaded at: https://www.mdpi.com/article/10.3390/ijms241813890/s1.
