# Supplementary material for: OncoUroMiR: Circulating miRNAs for Detection and Discrimination of the Main Urological Cancers Using a ddPCR-Based Approach
Source: Int J Mol Sci. 2023 Sep 9;24(18):13890. doi: 10.3390/ijms241813890 (PMC10531069; doi:10.3390/ijms241813890)
Supplement: Supplementary file 1 [file ijms-24-13890-s001.zip › ijms-2583554-supplementary.pdf]

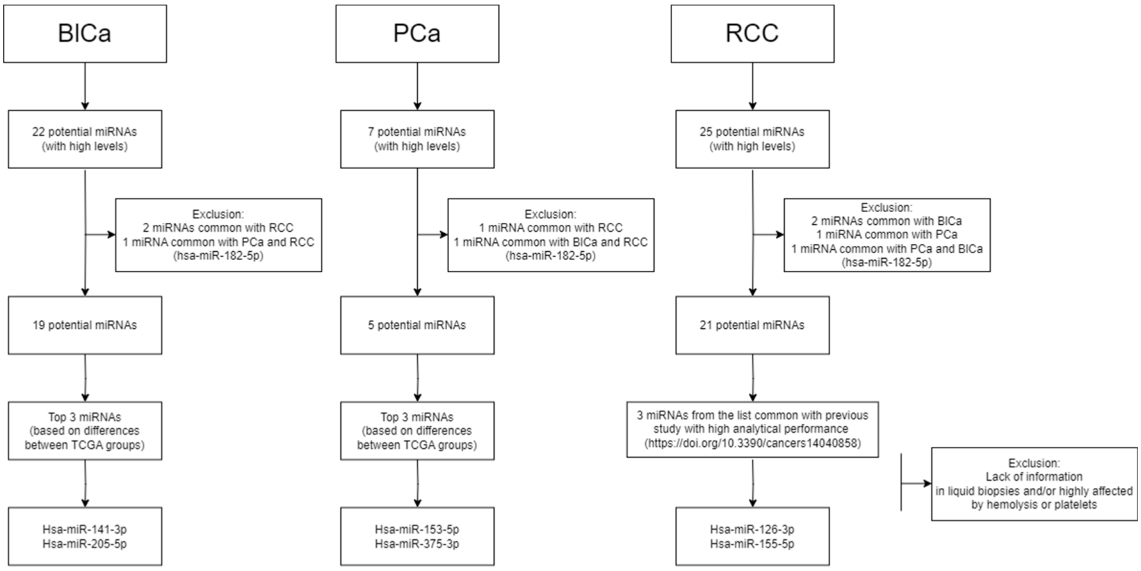

Figure S1. MiRNAs selection workflow through *in silico* analysis.

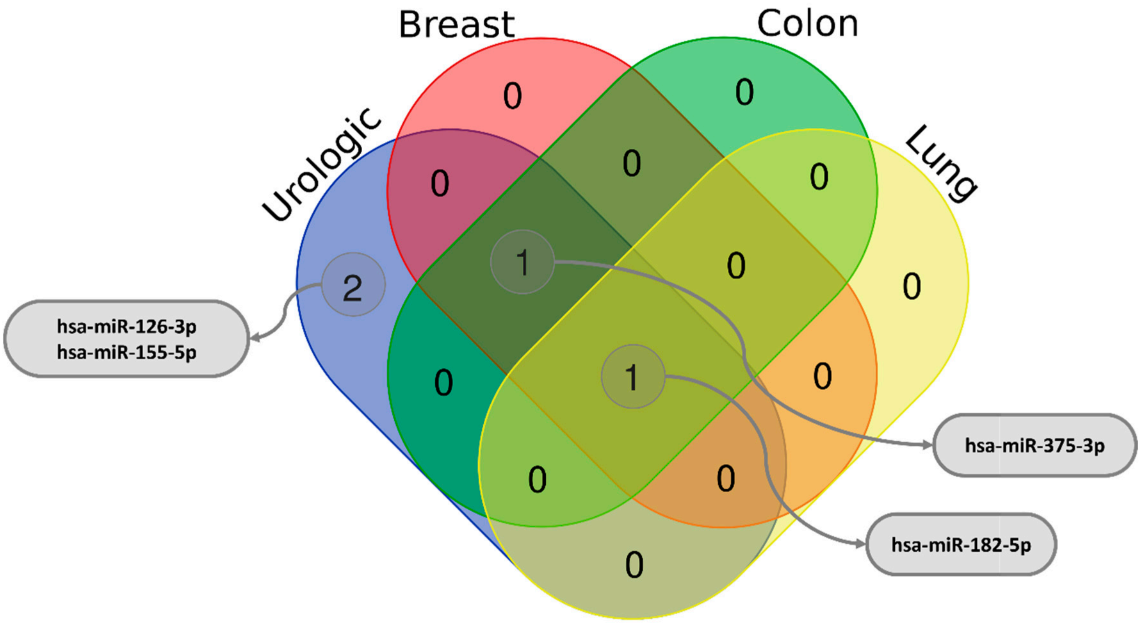

Figure S2. Venn diagram depicting the significantly different OncoUroMiR miRNAs in the studied urological cancers and in the three most common non-urological cancers. Venn diagram was constructed using a web-based tool (https://bioinformatics.psb.ugent.be/webtools/Venn/).

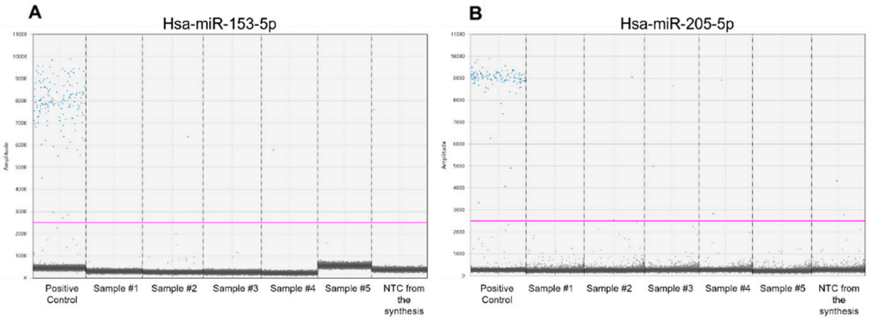

Figure S3. DdPCR 1D plots. A – Optimization of hsa-miR-153-5p. B – Optimization of hsa-miR-205-5p.

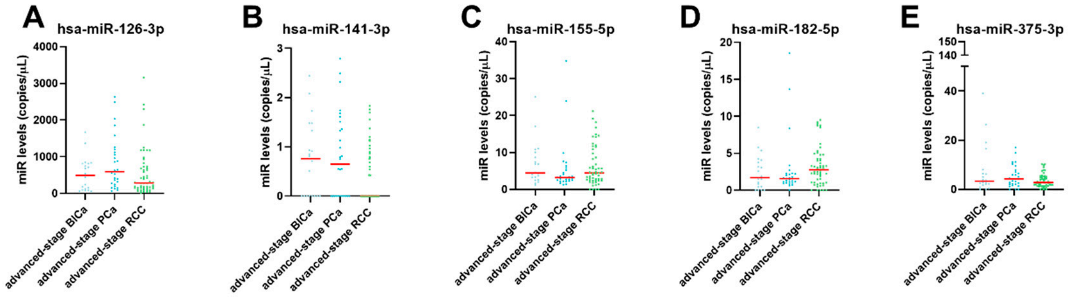

Figure S4. Dot-plots of hsa-miR-126-3p (A), hsa-miR-141-3p (B), hsa-miR-155-5p (C), hsa-miR-182-5p (D), and hsa-miR-375-3p (E) levels in advanced-stage urological cancers. The horizontal red lines represent miRNAs' median levels. Abbreviations: BlCa – Bladder Cancer, PCa – Prostate Cancer, RCC – Renal Cell Carcinoma.

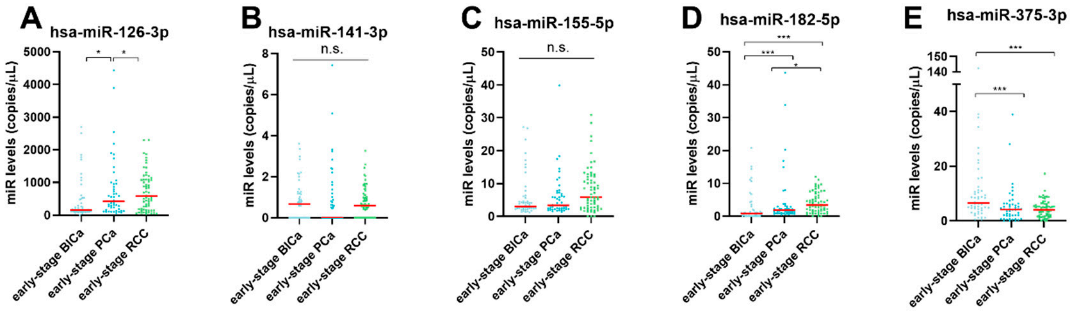

Figure S5. Dot-plots of hsa-miR-126-3p (A), hsa-miR-141-3p (B), hsa-miR-155-5p (C), hsa-miR-182-5p (D), and hsa-miR-375-3p (E) levels in early-stage urological cancers. The horizontal red lines represent miRNAs' median levels. Abbreviations: BlCa – Bladder Cancer, PCa – Prostate Cancer, RCC – Renal Cell Carcinoma, n.s. – not significant. * – p-value < 0.05, *** – p-value ≤ 0.001.

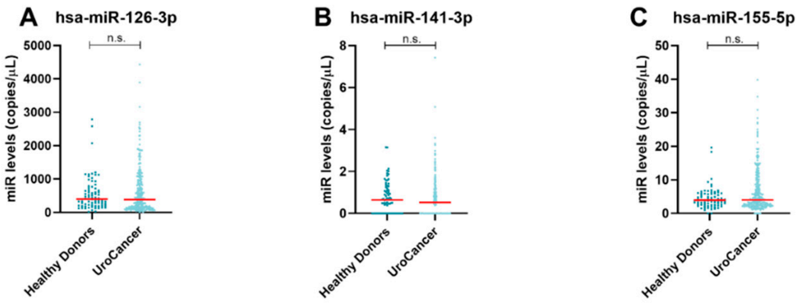

Figure S6. Dot-plots of hsa-miR-126-3p (A), hsa-miR-141-3p (B) and hsa-miR-155-5p (C) not significant levels in urological cancers and healthy donors. The horizontal red lines represent miRNAs' median levels. Abbreviations: n.s. – not significant.

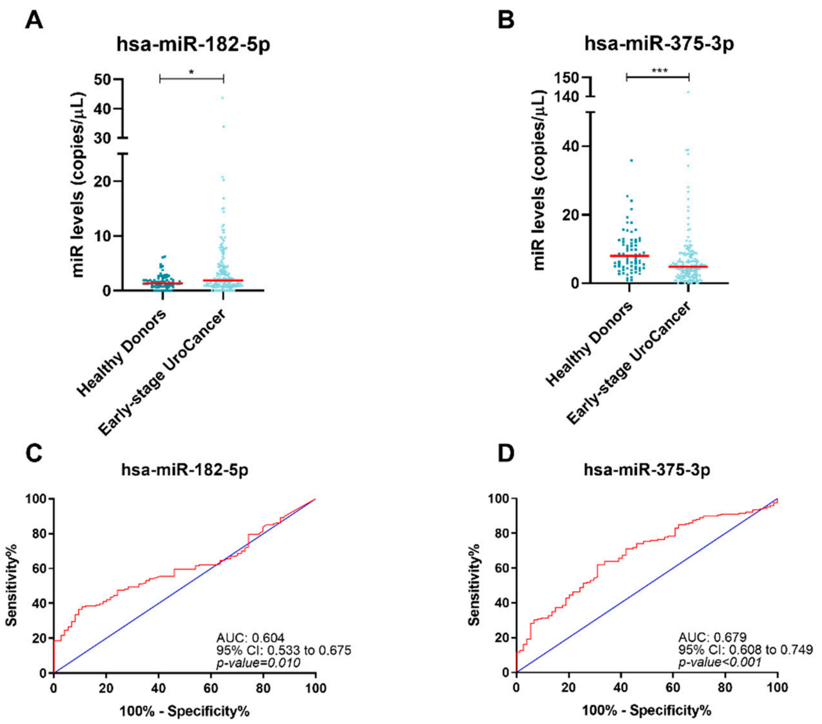

Figure S7. Dot-plots of hsa-miR-182-5p (A) and hsa-miR-375-3p (B) levels in early-stage urological cancers and Healthy Donors. The horizontal red lines represent miRNAs' median levels. Receiver operating characteristic curves (ROC) of hsa-miR-182-5p (C) and hsa-miR-375-3p (D) for early-stage urological cancers detection. The blue and red lines indicate the reference line and the identity line for each miRNA, respectively. * – *p*-value < 0.05, *** – *p*-value ≤ 0.001.

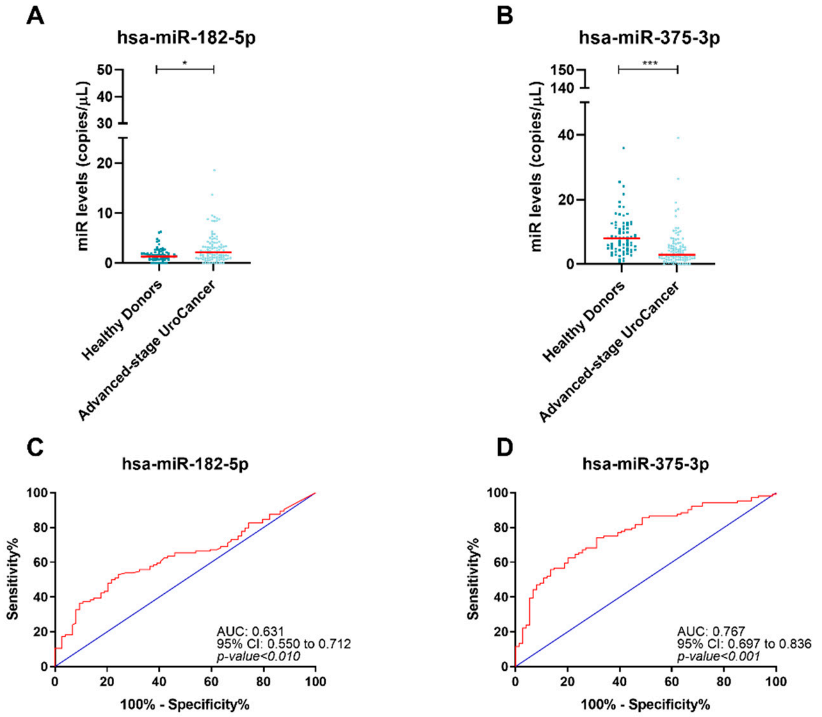

Figure S8. Dot-plots of hsa-miR-182-5p (A) and hsa-miR-375-3p (B) levels in advanced-stages urological cancers and Healthy Donors. The horizontal red lines represent miRNAs' median levels. Receiver operating characteristic (ROC) curves of hsa-miR-182-5p (C) and hsa-miR-375-3p (D) for advanced-stage urological

cancers detection. The blue and red lines indicate the reference line and the identity line for each miRNA, respectively. * – *p*-value ≤ 0.05, *** – *p*-value ≤ 0.001.

Table S1. Performance of hsa-miR-182-5p and hsa-miR-375-3p as biomarkers for early-stage Urological Cancers detection. Abbreviations: SE – Sensitivity; SP – Specificity; PPV – Positive Predictive Value; NPV – Negative Predictive Value.

miRNA	SE %	SP %	PPV %	NPV %	Accuracy %
**hsa-miR-182-5p**	36.75	90.54	89.71	38.95	53.33
**hsa-miR-375-3p**	62.05	68.92	81.75	44.74	64.17
**miRNA panel**	77.71	59.46	81.13	54.32	72.08

Table S2. Performance of hsa-miR-182-5p and hsa-miR-375-3p as biomarkers for advanced-stage Urological Cancers detection. Abbreviations: SE – sensitivity; SP – specificity; PPV – positive predictive value; NPV – negative predictive value.

miRNA	SE %	SP %	PPV %	NPV %	Accuracy %
**hsa-miR-182-5p**	37.25	90.54	84.44	51.15	59.66
**hsa-miR-375-3p**	73.53	68.92	76.53	65.38	71.59
**miRNA panel**	89.22	48.65	70.54	76.60	72.16

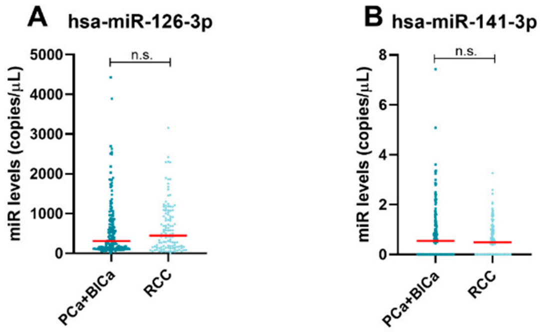

Figure S9. Dots-plots of hsa-miR-126-3p (A), and hsa-miR-141-3p (B) levels in RCC and BlCa/PCa group. The horizontal red lines represent miRNAs' median levels. Abbreviations: BlCa – Bladder Cancer, PCa – Prostate Cancer, RCC – Renal Cell Carcinoma, n.s. – not significant.

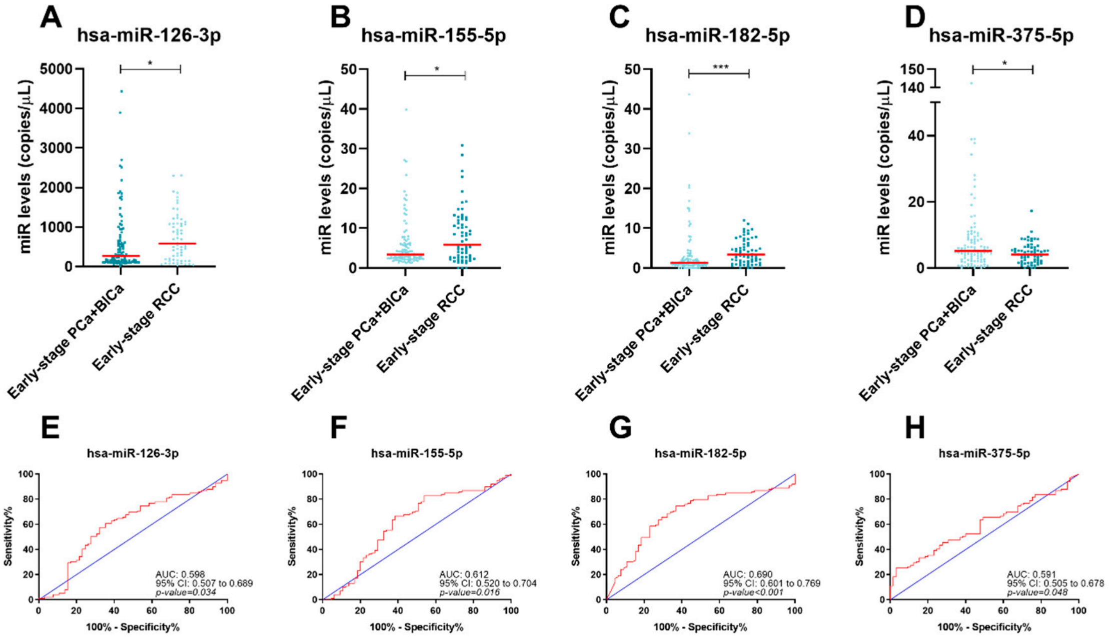

Figure S10. Dots-plots of hsa-miR-126-3p (A), hsa-miR-155-5p (B), hsa-miR-182-5p (C), and hsa-miR-375-3p (D) levels in early-stage RCC and early-stage BlCa/PCa group. The horizontal red lines represent miRNAs' median levels. Receiver operating characteristic (ROC) Curves of hsa-miR-126-3p (E), hsa-miR-155-5p (F), hsa-miR-182-5p (G), and hsa-miR-375-3p (H) for early-stage RCC detection. The blue and red lines indicate the reference line and the identity line for each miRNA, respectively. Abbreviations: BlCa – Bladder cancer, PCa – Prostate cancer, RCC – Renal cell carcinoma, * – p-value ≤ 0.05, *** – p-value ≤ 0.001.

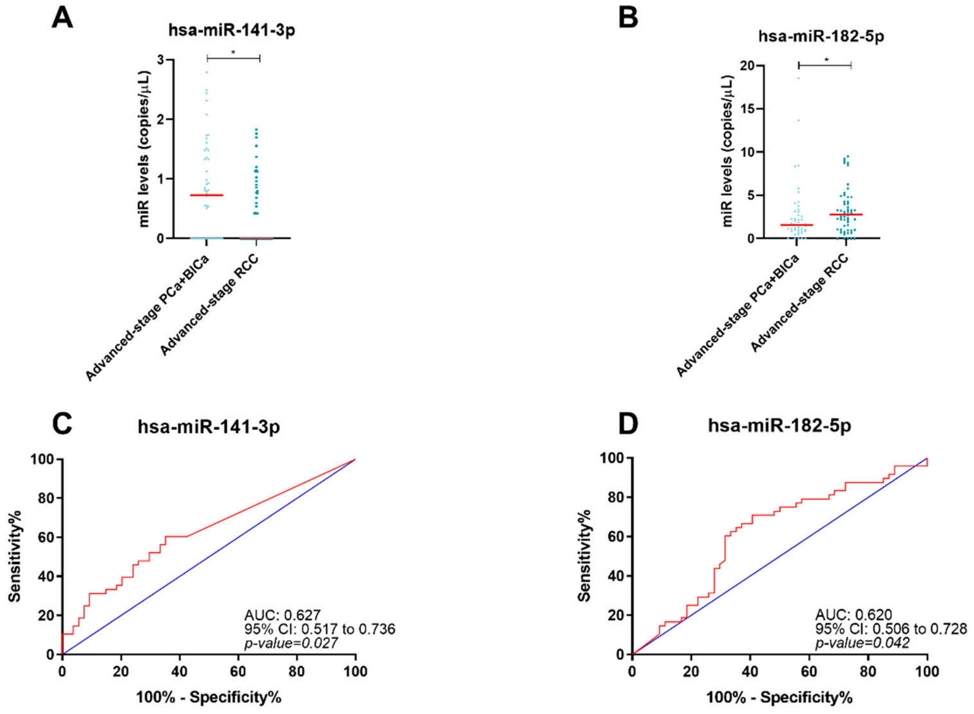

Figure S11. Dots-plots of hsa-miR-141-3p (A), and hsa-miR-182-5p (B) levels in advanced-stage RCC and BlCa/PCa group. The horizontal red lines represent miRNAs' median levels. Receiver operating characteristic (ROC) curves of hsa-miR-141-3p (C), and hsa-miR-182-5p (D) for advanced-stage RCC detection. The blue and red lines indicate the reference line and the identity line for each miRNA,

respectively. Abbreviations: BlCa – Bladder cancer, PCa – Prostate cancer, RCC – Renal cell carcinoma, * – p-value ≤ 0.05.

Table S3. Performance of hsa-miR-126-3p, hsa-miR-155-5p, hsa-miR-182-5p, and hsa-miR-375-3p as biomarkers for early-stage Urological Cancers detection. Abbreviations: SE – sensitivity; SP – specificity; PPV – Positive predictive value; NPV – Negative predictive value.

miRNA	SE %	SP %	PPV %	NPV %	Accuracy %
**hsa-miR-126-3p**	67.69	57.58	51.16	73.08	61.59
**hsa-miR-155-5p**	46.15	82.83	63.83	70.09	68.29
**hsa-miR-375-3p**	96.92	25.25	45.99	92.59	53.66
**hsa-miR-182-5p**	63.08	74.75	62.12	75.51	70.12
**miRNA panel (hsa-miR-155-5p + hsa-miR-375-3p)**	43.08	87.88	70.00	70.16	70.12

Table S4. Performance of hsa-miR-141-3p, and hsa-miR-182-5p as biomarkers for advanced-stage Urological Cancers detection. Abbreviations: SE – sensitivity; SP – specificity; PPV – Positive predictive value; NPV – Negative predictive value.

miRNA	SE %	SP %	PPV %	NPV %	Accuracy %
**hsa-miR-141-3p**	64.81	60.42	64.81	60.42	62.75
**hsa-miR-182-5p**	59.26	70.83	69.57	60.71	64.71

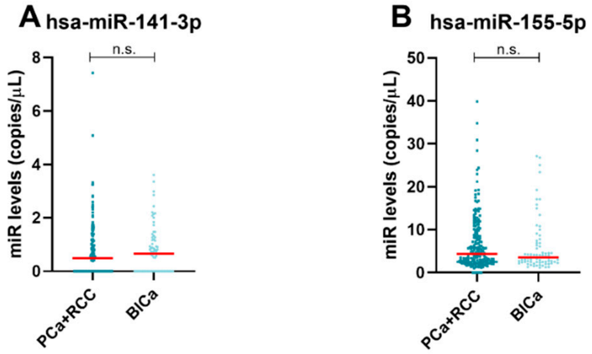

Figure S12. Dots-plots of hsa-miR-141-3p (A) and hsa-miR-155-5p (B) levels in BlCa and RCC/PCa group. The horizontal red lines represent miRNAs' median levels. Abbreviations: RCC – Renal Cell Carcinoma, BlCa – Bladder Cancer, PCa – Prostate Cancer, n.s. – not significant.

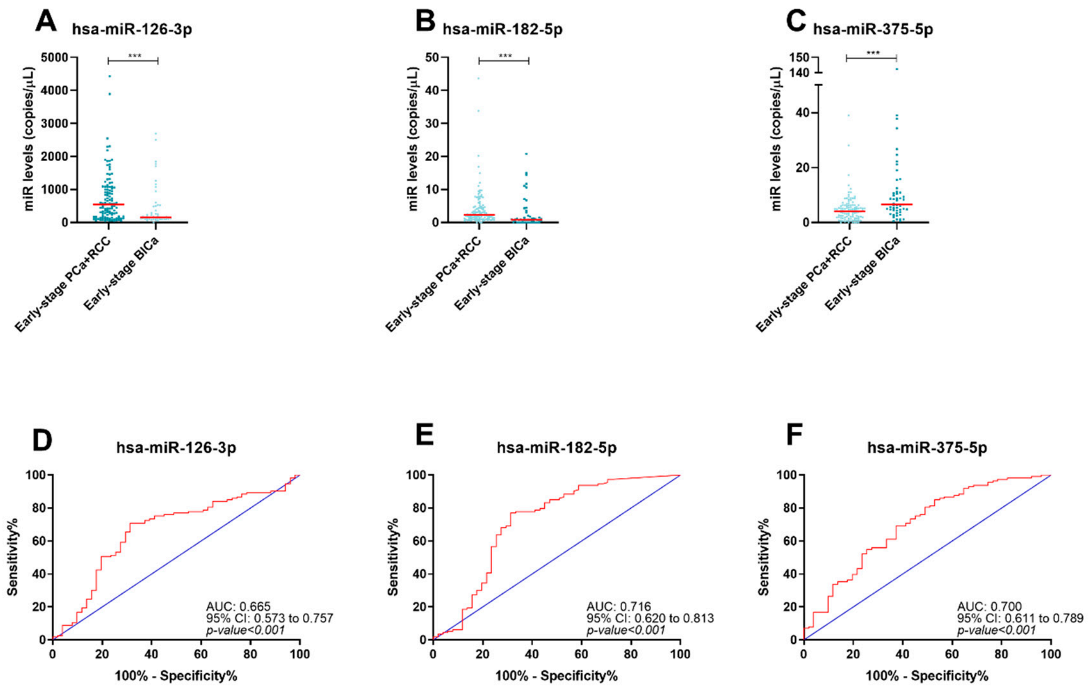

Figure S13. Dots-plots of hsa-miR-126-3p (A), hsa-miR-182-5p (B) and hsa-miR-375-3p (C) levels in early-stage BlCa and RCC/PCa group. The horizontal red lines represent miRNAs' median levels. Receiver operating characteristic (ROC) curves of hsa-miR-126-3p (D), hsa-miR-182-5p (E), and hsa-miR-375-3p (F) for early-stage BlCa detection. The blue and red lines indicate the reference line and the identity line for each miRNA, respectively. Abbreviations: BlCa – Bladder cancer, PCa – Prostate cancer, RCC – Renal cell carcinoma, *** – *p*-value ≤ 0.001.

Table S5. Performance of hsa-miR-126-3p, hsa-miR-182-5p, and hsa-miR-375-3p as biomarkers for early-stage BlCa detection. Abbreviations: SE – Sensitivity; SP – Specificity; PPV – Positive predictive value; NPV – Negative predictive value.

miRNA	SE %	SP %	PPV %	NPV %	Accuracy %
**hsa-miR-126-3p**	68.63	70.80	51.47	83.33	70.12
**hsa-miR-375-3p**	47.06	84.96	58.54	78.05	73.17
**hsa-miR-182-5p**	68.63	76.99	57.38	84.47	74.39
**miRNA panel (hsa-miR-126-3p + hsa-miR-375-3p)**	31.37	95.58	76.19	75.52	75.61

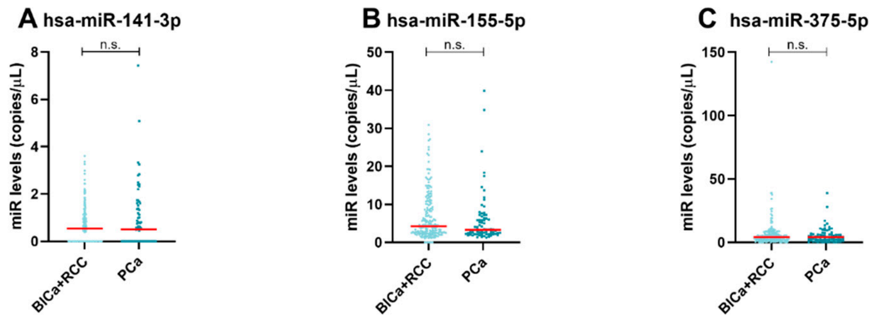

Figure S14. Dot-plots of hsa-miR-141-3p (A), hsa-miR-155-5p (B) and hsa-mIR-375-5p (C) levels in PCa and RCC/BlCa group. The horizontal red lines represent miRNAs' median levels. Abbreviations: BlCa – Bladder Cancer, PCa – Prostate Cancer, RCC – Renal Cell Carcinoma, n.s. – not significant.

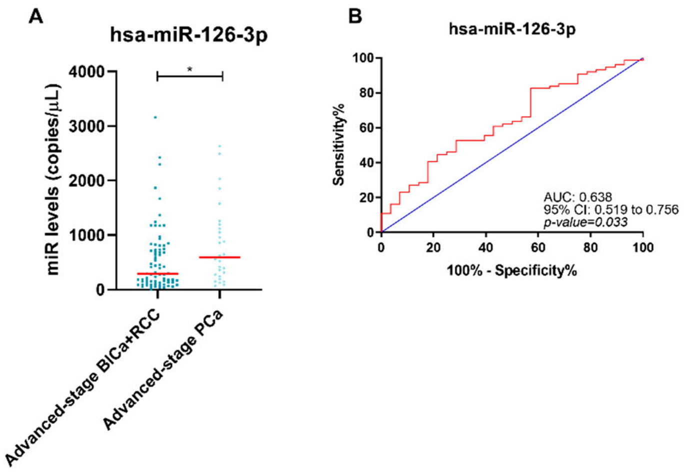

Figure S15. Dot-plot of hsa-miR-126-3p (A) in advanced-stage PCa and RCC/BlCa group. The horizontal red lines represent miRNAs' median levels. Receiver operating characteristic (ROC) curve of hsa-miR-126-3p (B) for advanced-stage PCa detection. The blue and red lines indicate the reference line and the identity line for each miRNA, respectively. Abbreviations: BlCa – Bladder cancer, PCa – Prostate cancer, RCC – Renal cell carcinoma, * – *p*-value ≤ 0.05.

Table S6. Performance of hsa-miR-126-3p for advanced-stage PCa detection. Abbreviations: SE – Sensitivity; SP – Specificity; PPV – Positive predictive value; NPV – Negative predictive value.

miRNA	SE %	SP %	PPV %	NPV %	Accuracy %
**hsa-miR-126-3p**	42.86	82.43	48.00	79.22	71.57

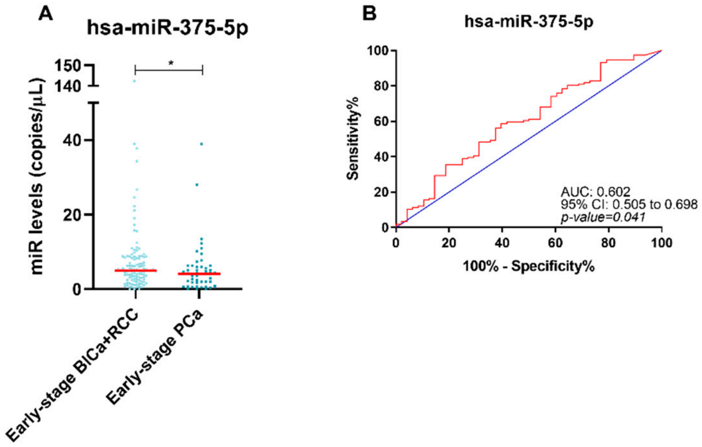

Figure S16. Dot-plot of hsa-miR-375-5p (A) in early-stage PCa and RCC/BlCa group. The horizontal red lines represent miRNAs' median levels. Receiver operating characteristic (ROC) curve of hsa-miR-375-5p (B) for early-stage PCa detection. The blue and red lines indicate the reference line and the identity line for each miRNA, respectively. Abbreviations: BlCa – Bladder cancer, PCa – Prostate cancer, RCC – Renal cell carcinoma, * – *p*-value ≤ 0.05.

Table S7. Performance of hsa-miR-375-5p for early-stage PCa detection. Abbreviations: SE – Sensitivity; SP – Specificity; PPV – Positive predictive value; NPV – Negative predictive value.

miRNA	SE %	SP %	PPV %	NPV %	Accuracy %
**hsa-miR-375-5p**	60.42	58.62	37.66	78.16	59.15

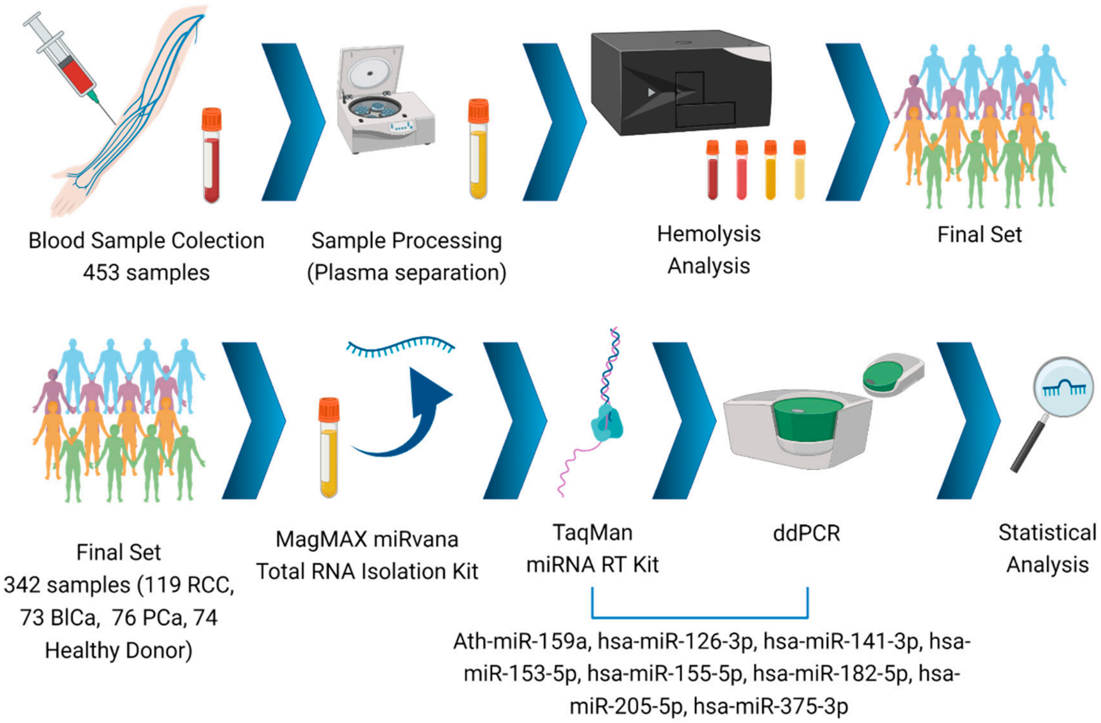

Figure S17. Methods' workflow of OncoUroMiR pipeline.
